# Prediction and Optimization of Surface Roughness for Laser-Assisted Machining SiC Ceramics Based on Improved Support Vector Regression

**DOI:** 10.3390/mi13091448

**Published:** 2022-09-01

**Authors:** Chen Cao, Yugang Zhao, Zhuang Song, Di Dai, Qian Liu, Xiajunyu Zhang, Jianbing Meng, Yuewu Gao, Haiyun Zhang, Guangxin Liu

**Affiliations:** School of Mechanical Engineering, Shandong University of Technology, Zibo 255049, China

**Keywords:** surface roughness, SiC ceramics, laser-assisted machining (LAM), optimization, GWO-SVR

## Abstract

In this paper, the surface roughness of SiC ceramics was investigated in laser-assisted machining (LAM) processes; machine learning was used to predict surface roughness and to optimize the process parameters, and therefore, to ultimately improve the surface quality of a workpiece and obtain excellent serviceability. First, single-factor turning experiments were carried out on SiC ceramics using LAM according to the material removal mechanism to investigate the variation trend of the effects of different laser powers, rotational speeds, feed rates, and cutting depths on surface roughness. Then, laser power, rotational speed, feed rate and cutting depth were selected as the four factors, and the surface roughness was used as the target value for the orthogonal experiments. The results of the single-factor experiments and the orthogonal experiments were combined to construct a prediction model based on the combination of the grey wolf optimization (GWO) algorithm and support vector regression (SVR). The coefficient of determination (*R*^2^) of the optimized prediction model reached 0.98676 with an average relative error of less than 2.624%. Finally, the GWO algorithm was used to optimize the global parameters of the prediction model again, and the optimal combination of process parameters was determined and verified by experiments. The actual minimum surface roughness (*Ra*) value was 0.418 μm, and the relative error was less than 1.91% as compared with the predicted value of the model. Therefore, the prediction model based on GWO-SVR can achieve accurate prediction of the surface roughness of SiC ceramics in LAM and can obtain the optimum surface roughness using parameter optimization.

## 1. Introduction

With the advancement of science and technology, the production process of engineering ceramics has improved, and the high hardness, physical stability, thermal resistance, chemical inertness, and biocompatibility of the materials can meet the demanding requirements of different environments of service [1,2,3]. Engineering ceramics are widely used in aerospace, automotive, biomaterial, communication, and defence applications for the production of rocket nozzle parts, automotive engine valves and brake pads, prosthetic bones, semiconductors, and turbine blades [4,5,6]. Grinding is the main traditional processing method for ceramics, but it is not efficient enough to meet the needs of the industry and surface integrity needs to be optimized, especially in terms of surface roughness [7]. With the growth of the laser processing market, LAM methods have reduced processing costs by approximately 60–80% and have significantly improved the surface quality of machined alumina ceramics as compared with conventional diamond grinding [8,9].

Surface roughness is one of the key evaluation indicators of surface integrity and is closely related to a component’s assembly properties, wear resistance, fatigue strength, contact stiffness, vibration, and noise. Reasonable surface roughness gives mechanical components good serviceability, while excessive improvements in surface roughness can result in increased machining costs. Therefore, the prediction of surface roughness has aroused much interest, and the construction of accurate roughness prediction models to obtain the optimal machining condition has become a hot research topic. Previously, researchers have selected different materials as research objects and used different research strategies to complete the work on surface roughness prediction and optimization. Deng et al. established a prediction model for the surface roughness of chemical mechanical polishing using back propagation (BP) neural networks. The surface roughness decreased with increasing abrasive size and abrasive concentration, increased with increasing polishing pressure, and decreased and then increased with increasing polishing speed, and the model accurately predicted the relationship between process parameters and surface roughness, which provided theoretical guidance for chemical mechanical polishing of silicon carbide substrates [10]. Ting et al. established artificial neural network (ANN), support vector machine (SVM), and regression analysis (RA) models to predict machined surface roughness of titanium alloy. The artificial neural network model had the best prediction accuracy because of its lowest relative error between the predicted and experimental values [11]. Maher et al. used an adaptive neuro-fuzzy inference system to establish a prediction model of surface roughness using milling parameters and cutting forces to achieve accurate prediction of cutting force signals; the average prediction accuracy reached 96.65% [12]. Touggui et al. used the response surface methodology to establish quadratic regression models of surface roughness and material removal rate to study the effects of cutting speed, feed, and cutting depth to achieve accurate prediction of surface roughness and material removal rate as well as desirability function optimization [13]. Sekulic et al. investigated the relationships among four process parameters, namely, spindle speed, feed per tooth, axial depth of cut and radial depth of cut, and surface roughness for ball milling of hardened steel by comparing the prediction accuracy of the response surface method genetic algorithm and grey wolf optimization algorithm for surface roughness, which was determined to have the highest prediction accuracy with the training and test set accuracy reaching 91.8% and 89.58% respectively [14]. Manjunath Patel et al. investigated four process parameters affecting surface roughness, namely, cutting speed, feed rate, depth of cut, and tool tip radius, in a central composite design experiment for high-strength aluminium alloy turning, and the regression model established was optimized by principal component analysis and the JAYA algorithm with an absolute deviation of surface roughness of 7.97% [15]. Karim et al. investigated the effects of cutting speed, feed rate, and cutting depth on the surface roughness of turning silicon carbide-reinforced aluminium alloy composites by conducting orthogonal experiments, which were optimized based on the Taguchi experiment of signal-to-noise ratio and principal component analysis. The ANOVA showed that the cutting speed and feed rate were the most significant factors affecting surface roughness, and the MAPE value of roughness was within 0.23% with a correlation coefficient of 0.98286, which verified the validity of the experiment; the final optimization results of the principal component analysis method showed that when the cutting speed, feed rate, and cutting depth were 396 m/min, 0.16 mm/rev, 0.85 mm, a surface roughness of 0.7257 μm could be obtained [16]. Patel et al. proposed the use of image processing, computer vision, and machine learning for surface roughness prediction to achieve online inspection and qualitative assessment of machined parts. The prediction results of two algorithms, bagging tree and stochastic gradient boosting were compared and the results showed that the correlation coefficient of the bagging tree algorithm was 0.9289 in ten-fold cross-validation, and the prediction results were more reliable [17]. Li et al. first collected temperature and vibration monitoring data using thermocouples, infrared temperature sensors, and accelerometers for the additive manufacturing process. Then, they extracted time and frequency domain features from the monitoring data and trained a surface roughness prediction model using an integrated learning algorithm, and finally verified the validity of the model by using additive manufacturing monitoring data on a fused filament manufacturing machine to achieve high accuracy in predicting the surface roughness of 3D printed parts [18].

The continuous research on surface roughness prediction has shown that, with the rapid development of artificial intelligence, modeling methods that combine the experimental design method with artificial intelligence optimization are becoming more and more popular. The prediction of surface roughness is a complex nonlinear problem, and the prediction model cannot be established by ordinary linear regression. Quadratic regression has the shortcomings of overtraining and overfitting. A support vector machine (SVM) model has strong nonlinear fitting function and excellent generalization ability for small datasets, which is widely used in pattern recognition, classification prediction, and regression problems [19]. The GWO algorithm is the latest metaheuristic algorithm with a simple principle, few control parameters, and the ability to rapidly converge to achieve global optimization [20,21]. LAM of ceramic processes is also a hot research topic worldwide, but there is a lack of research data on turning SiC ceramics. As compared with the SVM model, this hybrid optimization algorithm, GWO-SVR, has the advantages of high diagnostic accuracy and strong generalization capability.

Therefore, in this paper, we describe the principle of turning SiC ceramics and the material removal mechanism based on LAM technology. First, we investigate the effects of laser power, rotational speed, feed, and cutting depth on surface roughness through single-factor experiments. Then, we construct a surface roughness prediction model based on GWO-SVR using orthogonal experimental results, and finally use the GWO algorithm again to determine the optimal combination of process parameters to provide guidance for the precision machining of SiC ceramics.

## 2. Materials and Methods

### 2.1. Experimental Material and Equipment

The SiC ceramic rod samples were prepared by pressureless sintering with a size of Φ11 × 45 mm, and were provided by Guangdong XY Fine Ceramic Technology Co., Ltd. (Dongguan, China). The material property parameters are shown in Table 1.

LAM of SiC ceramics has high requirements for tool performance, both in terms of high strength and hardness of the ceramics to tool wear; high temperatures decrease tool hardness and the bonding phenomenon with the ceramic material is caused by increasing the temperature. The choice of tool is important to the quality of the machined surface and the cost of machining. Cubic boron nitride (CBN) has a high hardness, which can reach 3000–5000 HV, second only to diamond, with good thermal hardness, wear resistance, excellent thermal stability, excellent chemical stability, and a low coefficient of friction. Its hardness and chemical stability enable the tool to withstand the high thermal and mechanical loads generated during hard turning operations, which are suitable for machining hard and difficult to machine materials [22,23,24,25]. Therefore, a CBN insert (CNGA120408 FBS7000) and a matching toolholder (MCLN2020K12) were selected for LAM of SiC ceramics.

**Figure 1:**
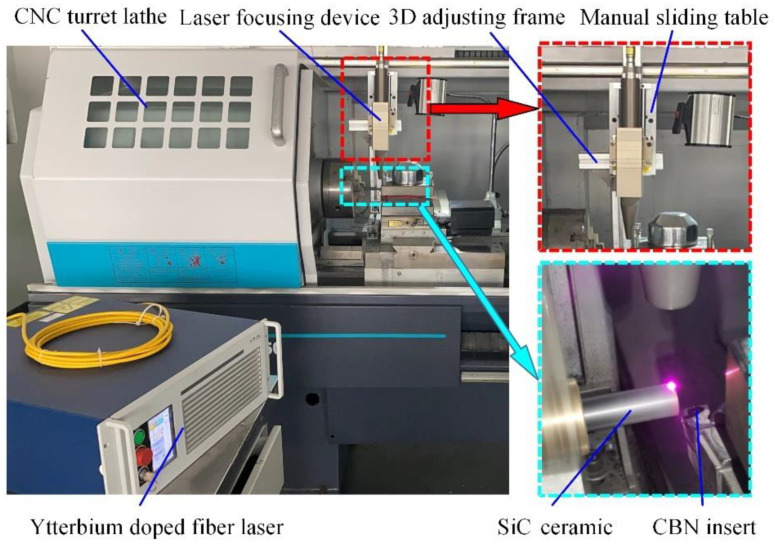
Layout of experimental equipment.

As shown in Figure 1, the experimental platform used in this study consisted of two main components, i.e., the laser-assisted heating device and the processing equipment. The laser-assisted heating device (YLR-150/1500-QCW-MM-AC, IPG Photonics Co., Oxford, MI, USA) consists of an ytterbium-doped laser and a focusing device that uses a continuous laser with a maximum average power of 250 W. The ytterbium-doped fiber laser emits a laser that travels through the fiber, and then passes through the focusing device to produce a high-energy laser beam that irradiates vertically onto the workpiece surface. The processing equipment was a CNC turret lathe (CKD6136i, Dalian Machine Tools Group, Dalian, China) with a FANUC operating system, a four-station automatic tool holder, and a 3D adjusting frame. The laser focusing device was mounted on the 3D adjusting frame, which allowed for position adjustments in three coordinate directions; the 3D adjusting frame was mounted on the lathe bedsaddle and followed the synchronous movement of the bedsaddle.

### 2.2. Processing Principle and Material Removal Mechanism

**Figure 2:**
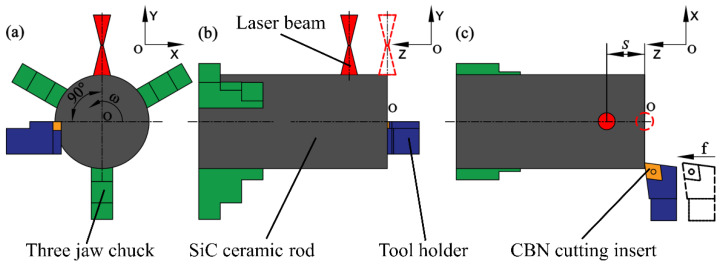
Schematic diagram of LAM (**a**) Right view, (**b**) Main view, (**c**) Top view.

As shown in Figure 2, the coordinate system is fixed with the center of the circle on the right end face of the workpiece as the origin and the workpiece rotates uniformly at an angular velocity *ω* around the Z axis. The laser beam vertically irradiates the surface of the ceramic rod. The horizontal distance from the center point of the laser beam to the tool tip is *S*, which is the preheating length of LAM. After the start of the CNC machining program, the laser beam and the tool both move from the starting position (marked by the red dashed line and the black dashed line) and synchronously at a feed speed *f* along the *Z*-axis for a distance *S*. At this time, the tool tip is exactly in contact with the end face of the workpiece and the preheating is over; then, the laser beam and the tool continue to move synchronously at the programmed feed speed to achieve laser-assisted turning of SiC ceramics. Combined with the temperature field simulation and exploratory experiments, it was determined that *S* = 1 mm and a preheating time of 60 s could achieve a good preheating effect.

The brittle removal and plastic removal methods directly affect the surface quality of the workpiece and the cost of manufacturing, therefore, the material removal mechanism was analyzed here in conjunction with material fracture theory. The yield strength of SiC ceramics is much higher than the fracture strength at low temperature. The brittle fracture occurs in the shear zone of the material with the crack propagating inside the grain, and the transgranular fracture occurs, resulting in fragment chips. With the laser-assisted heating, the ceramic temperature increases gradually, the brittle-ductile transition occurs in the material, and the yield strength decreases gradually until it is lower than the fracture strength [26,27,28]. There are two forms of brittle and ductile fracture in the material. The crack propagates in the grain interior and along the grain boundary, and transgranular and intergranular fractures occur, resulting in granular and arc chips. When the temperature continues to rise close to the melting point, the yield strength is far lower than the fracture strength. The crack propagates along the grain boundary, and the intergranular fracture occurs, resulting in arc chips and even continuous chips.

Ravindra et al. studied the hardness and yield strength of SiC ceramics [29]. The hardness of SiC is close to 26 GPa at room temperature. When the temperature reaches 1400 °C, the yield strength of SiC is 4.02 GPa, slightly larger than the fracture strength of the material in Table 1 The machinability is poor due to the hardness value of 8.835 GPa, which not only causes poor surface quality, but also increases the cost of tool wear. When the temperature reaches 1500 °C, the yield strength is 3.09 GPa, which is less than the fracture strength of the material. The hardness decreases to 6.8 GPa, and the softening degree of the material increases, making it easier for CBN tool cutting.

## 3. Experiments and Modeling

### 3.1. Effect of Factors on Surface Roughness

In order to investigate the effect of various process parameters on the surface roughness *Ra* value of SiC ceramics with single-factor experiments in LAM, the surface of the specimen was ultrasonically cleaned after machining using anhydrous ethanol to remove surface impurities. The surface roughness of three areas of the machined surface were randomly measured using a 3D digital microscope (DSX1000, OLYMPUS, Tokyo, Japan), and the average value was taken as the final surface roughness. The ranges of the process parameters are shown in Table 2, with values of 205 W, 1620 r/min, 3 mm/min, and 0.15 mm specified for the standard experimental conditions.

#### 3.1.1. Effect of Laser Power on Surface Roughness

**Figure 3:**
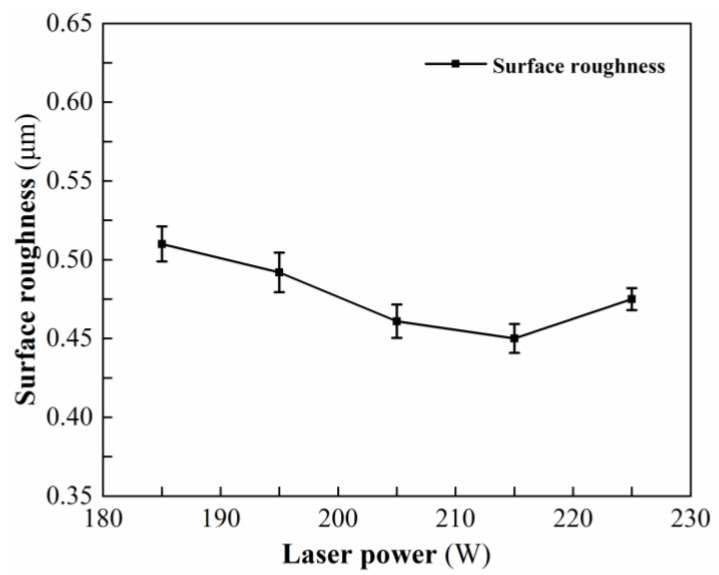
Effect of laser power on surface roughness.

Table 3 shows the measured results of surface roughness at different laser powers. Figure 3 shows the variation trend of surface roughness under different laser powers. When the laser power is 215 W, the minimum surface roughness is 0.437 μm; when the laser power is less than 215 W, as the laser power increases, the surface temperature of the workpiece is higher, the hardness is lower, the depth of the softening layer is deeper, and the plastic removal degree of the material is higher. The defects affecting the surface roughness such as pits and dents are gradually reduced, the surface roughness is gradually reduced, and the surface quality is better and better. When the laser power increases from 215 W to 225 W, the higher the surface temperature of the workpiece, the larger the depth of the softening layer, resulting in thermal damage on the machined surface and larger surface roughness. If the laser power continues to increase, the temperature gradient becomes too large, and the surface thermal stress exceeds the critical value of SiC ceramics, resulting in subsurface cracks. In addition, the subsurface cracks gradually propagate to the surface layer, resulting in material spalling, thermal burns, and ultimately a sharp increase in surface roughness.

#### 3.1.2. Effect of Rotational Speed on Surface Roughness

**Figure 4:**
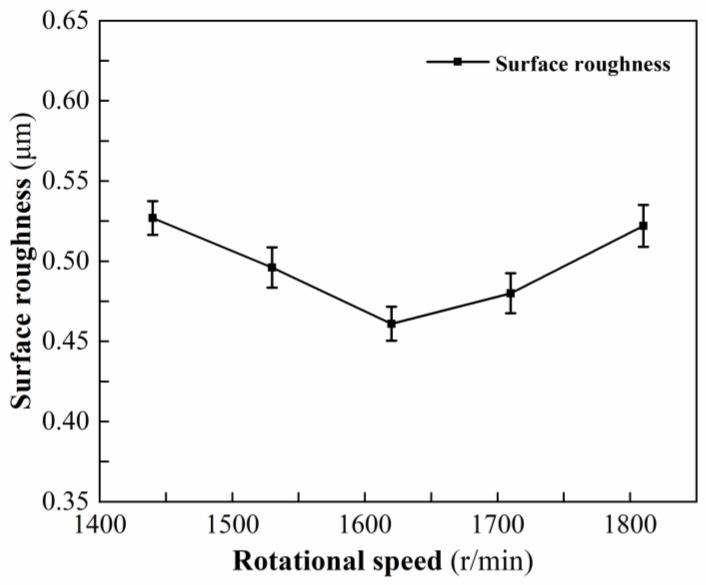
Effect of rotational speed on surface roughness.

Table 4 shows the measured results of surface roughness at different rotational speeds. Figure 4 shows the variation trend of surface roughness under different rotational speeds. When the rotational speed is 1620 r/min, the minimum surface roughness is 0.461 μm. When the rotational speed gradually increases, the energy distribution is more uniform, but for each turn of the workpiece, the preheating time of the same area on the workpiece surface decreases, and the temperature gradient decreases, resulting in a decrease in the softening degree. Therefore, when the rotational speed is less than 1620 r/min, with an increase in the rotational speed, the surface temperature of the laser preheating area decreases gradually, the depth of the softening layer is smaller, the degree of thermal damage of the material is gradually reduced to the state suitable for plastic processing, the defects affecting the surface roughness such as pits are gradually reduced, the surface roughness is gradually reduced, and the surface quality becomes better and better. When the rotational speed increases from 1620 r/min to 1800 r/min, the temperature of the laser preheating area becomes lower and lower, the softening degree decreases, the depth of the softening layer decreases, and the processing state transforms from plastic processing to brittle processing state. An increase in the brittle removal ratio of the material causes the surface roughness to become lager.

#### 3.1.3. Effect of Feed Speed on Surface Roughness

**Figure 5:**
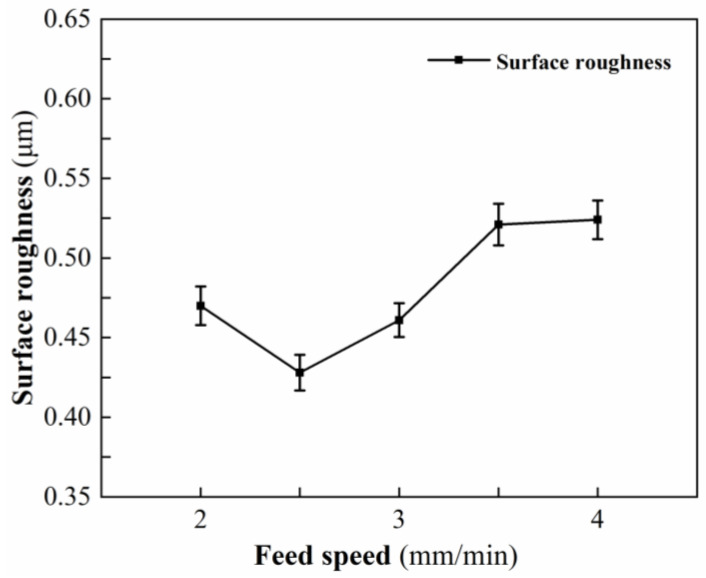
Effect of feed speed on surface roughness.

Table 5 shows the measured results of surface roughness at different feed speeds. Figure 5 shows the variation trend of surface roughness at different feed speeds. When the feed speed is 2.5 mm/min, the minimum surface roughness is 0.428 μm. When the feed speed increases from 2 mm/min to 2.5 mm/min, the preheating time in the same area of the workpiece surface decreases, the temperature gradually decreases, the degree of softening is more suitable for plastic processing, and defects such as tiny pits and dents caused by thermal damage gradually decrease or even disappear, resulting in a gradual decrease in surface roughness. When the feed speed increases from 2.5 mm/min to 4 mm/min, the temperature of the laser preheating area continues to decrease, the degree of softening is insufficient, the processing state transforms from plastic to brittle, and the height of the material residue on the workpiece surface in the feed direction increases, resulting in a larger surface roughness value.

#### 3.1.4. Effect of Cutting Depth on Surface Roughness

**Figure 6:**
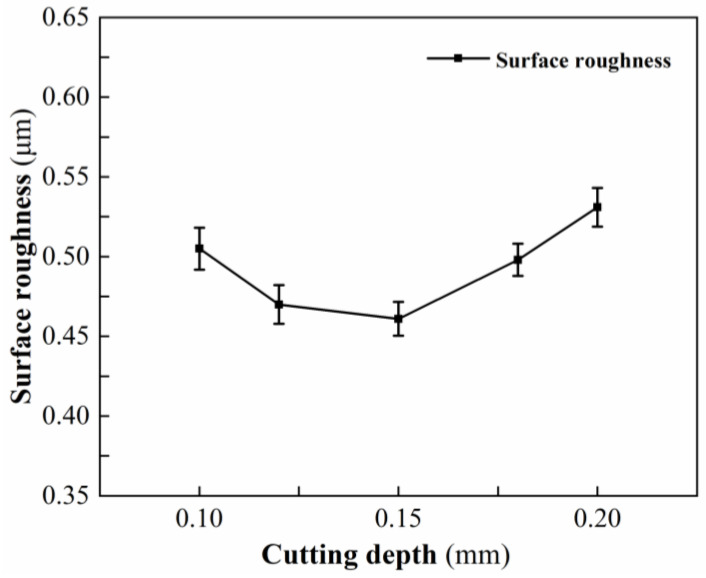
Effect of cutting depth on surface roughness.

Table 6 shows the measured results of surface roughness at different cutting depths. Figure 6 shows the variation trend of surface roughness at different cutting depths. When the cutting depth is 0.15 mm, the minimum surface roughness is 0.461 μm. As the cutting depth increases from 0.1 mm to 0.15 mm, the temperature of the laser preheating area gradually decreases, the degree of softening becomes more suitable for plastic processing, the defects caused by thermal damage gradually decrease, and the surface roughness gradually decreases. When the cutting depth increases from 0.15 mm to 0.2 mm, the temperature of the laser preheating area continues to decrease, the degree of softening is insufficient, the material removal method transforms to brittle removal, while an increase in cutting depth causes an increase in cutting force, tool wear, and tool vibration, resulting in an increase in the number and depth of scratches on the surface of the workpiece, and even the appearance of crater defects, which eventually lead to a larger surface roughness.

### 3.2. Ra Value Prediction Model Construction of Surface Roughness

#### 3.2.1. Orthogonal Experimental Design and Results

Based on the results of the single-factor experiments, the effect of each factor on surface roughness was studied. In order to achieve an accurate prediction of surface roughness, a prediction model based on SVR needed to be constructed. The orthogonal experiment not only achieved the collection of sample data required for the model, but also saved time and improved experimental efficiency. According to the effect of a single factor, the levels of each factor are determined, as shown in Table 7.

The orthogonal experimental scheme selects the L9(3^4^) experimental combination with the surface roughness *Ra* value as the response term, and the results are shown in Table 8.

#### 3.2.2. SVR Prediction Model

Vapnik et al. proposed the SVM algorithm based on machine learning [30], which could realize data classification or regression prediction, and successfully applied it to biology, machinery, and other fields [31,32]. For nonlinear problems, the SVR model can map nonlinear regression problems in original data sample space to linear regression problems in high-dimensional space. The optimal hyperplane is determined by maximizing the classification interval, and the linear regression of data is realized. Finally, the required optimal solution is obtained by analysis.

The results of the single-factor experiments as well as the orthogonal experiments in the paper are sorted to obtain Table 9 with the remove of the duplicate process parameter combinations.

Each group of process parameters and surface roughness *Ra* values are selected as the input and output of the original data sample space, which can be expressed as: (***x****_i_*, *Ra_i_*), (*i* = 1, 2, 3, …, 24, 25), where ***x****_i_* is the input feature vector, namely, the combination of process parameters and *Ra_i_* is the surface roughness *Ra* value output under the corresponding combination of process parameters.

The linear regression prediction model constructed in high-dimensional space is:

(1) $$y_{Ra}=\omega \cdot \varphi (x)+a$$

where φ(x) is the mapping function from the input to the target output, ω is the regression coefficient of φ(x), and a is the bias.

According to the structural risk minimization criterion, the optimized objective function and constraint conditions are transformed into the following form:

(2) $$\left\{\begin{array}{l} min\frac{1}{2}{\left\|\omega \right\|}^{2}+C\sum_{i=1}^{25}\left(\xi_{i}+\xi_{i}{}^{*}\right)\\ s.t.\left\{\begin{array}{l} y_{i}-\omega \cdot \varphi (x)-a\leq \varepsilon +\xi_{i}\\ \omega \cdot \varphi (x)+a-y_{i}\leq \xi_{i}{}^{*}+\varepsilon \\ \xi_{i},\xi_{i}{}^{*}\geq 0,i=1,2,...25 \end{array}\right. \end{array}\right.$$

where ${\left\|\omega \right\|}^{2}$ is the penalty function, C is the penalty coefficient, and ε is the insensitive loss function. When the distance between the sample space and the prediction model is less than ε, the sample space does not include the loss. $\xi_{i}$ and $\xi_{i}{}^{*}$ are the upper and lower limit of relaxation factor, respectively, and $y_{i}$ is the target output value.

In order to avoid the complexity of operations in high-dimensional space, the kernel function $K(x_{i},x_{j})=\varphi (x_{i})\cdot \varphi (x_{j})$ is introduced, transforming operations in high-dimensional space into operations in the original space. The radial basis function can improve the fitting accuracy and reduce the prediction error due to its nonlinear mapping and fast convergence. Therefore, the Gaussian radial basis kernel function (Equation (3)) is selected as the kernel function in this study:

(3) $$K(x_{i},x_{j})=\exp\left(-\frac{{\left\|x_{i}-x_{j}\right\|}^{2}}{2\sigma^{2}}\right)$$

where *σ* is the radius parameter of the kernel function and b determines the radial range of the kernel function action.

By solving the above equations, the regression function for the surface roughness SVR prediction model can be derived as Equation (4):

(4) $$y_{Ra}=\sum_{i=1}^{25}\left(\alpha_{i}-\beta_{i})\cdot K(x_{i},x_{j}\right)+a$$

where $\alpha_{i}$ and b $\beta_{i}$ are Lagrangian operators.

#### 3.2.3. GWO Algorithm

The penalty coefficient *C* of SVR balances the complexity of the regression model and the magnitude of the error, which affects the model training error. The insensitive loss function *ε* affects the generalization ability and prediction accuracy of the regression model. Therefore, the selection of the internal parameters (*ε*, *C*) of SVR determines the prediction performance of the model. In order to construct a better surface roughness prediction model, this study optimizes *ε* and *C* by using the GWO algorithm.

In the optimization process of the grey wolf algorithm, the grey wolf population contains four classes; *α*, *β,* and *δ* wolves form the dominant class of the wolf group, representing the three optimal solutions, respectively, and the remaining wolves are labeled as ω, as candidate solutions. The main steps of the GWO algorithm include calculating the distance between wolf individuals and updating the offspring wolf individuals, as shown in Equations (5) and (6), respectively:

(5) $$D=\left|C\cdot X_{p}\left(t\right)-X\left(t\right)\right|$$

(6) $$X\left(t+1\right)=X_{p}\left(t\right)-A_{1}\cdot D$$

where *A* and *C* are coefficient vectors as shown in Equations (7)–(9):

(7) $$A=2b\cdot r_{1}-b$$

(8) $$b=2-t/t_{max}$$

(9) $$C=2r_{2}$$

where *b* is a linearly decreasing convergence factor within [2, 0]; $r_{1}$, $r_{2}$ takes a random vector between [0, 1]; t is the number of current iterations; and $t_{max}$ is the maximum number of iterations.

The hunting process includes wolf leaning, predation Equations (10) to (12) and updating of offspring positions Equations (13) to (16):

(10) $$D_{1}=\left|C_{1}\cdot X_{\alpha }\left(t\right)-X_{\omega }\left(t\right)\right|$$

(11) $$D_{2}=\left|C_{2}\cdot X_{\beta }\left(t\right)-X_{\omega }\left(t\right)\right|$$

(12) $$D_{3}=\left|C_{3}\cdot X_{\delta }\left(t\right)-X_{\omega }\left(t\right)\right|$$

(13) $$X_{1}=X_{\alpha }\left(t\right)-A_{1}\cdot D_{\alpha }$$

(14) $$X_{2}=X_{\beta }\left(t\right)-A_{2}\cdot D_{\beta }$$

(15) $$X_{3}=X_{\delta }\left(t\right)-A_{3}\cdot D_{\delta }$$

(16) $$X\left(t+1\right)=(X_{1}+X_{2}+X_{3})/3$$

where $X_{\alpha }\left(t\right)$, $X_{\beta }\left(t\right)$, $X_{\delta }\left(t\right)$, and $X_{\omega }\left(t\right)$ are the current positions of wolves *α*, *β*, *δ* and *ω*, respectively; $D_{1}$, $D_{2}$, and $D_{3}$ are the distances between individuals of three classes *α*, *β,* and *δ,* and *ω*, respectively; $C_{1}$, $C_{2}$ and $C_{3}$ denote the random perturbations to wolves *α*, *β,* and *δ*, respectively; $X_{1}$, $X_{2}$ and $X_{3}$ denote the updated positions of *α*, *β,* and *δ* after guidance to other wolves, respectively; $X\left(t+1\right)$ is the final optimal search position of the offspring grey wolves. When $\left|A\right|<1$, the iterative convergence condition is satisfied, the prey position is successfully searched, and the optimal solution of the optimization objective is finally obtained.

#### 3.2.4. Surface Roughness Prediction Model for GWO-SVR

**Figure 7:**
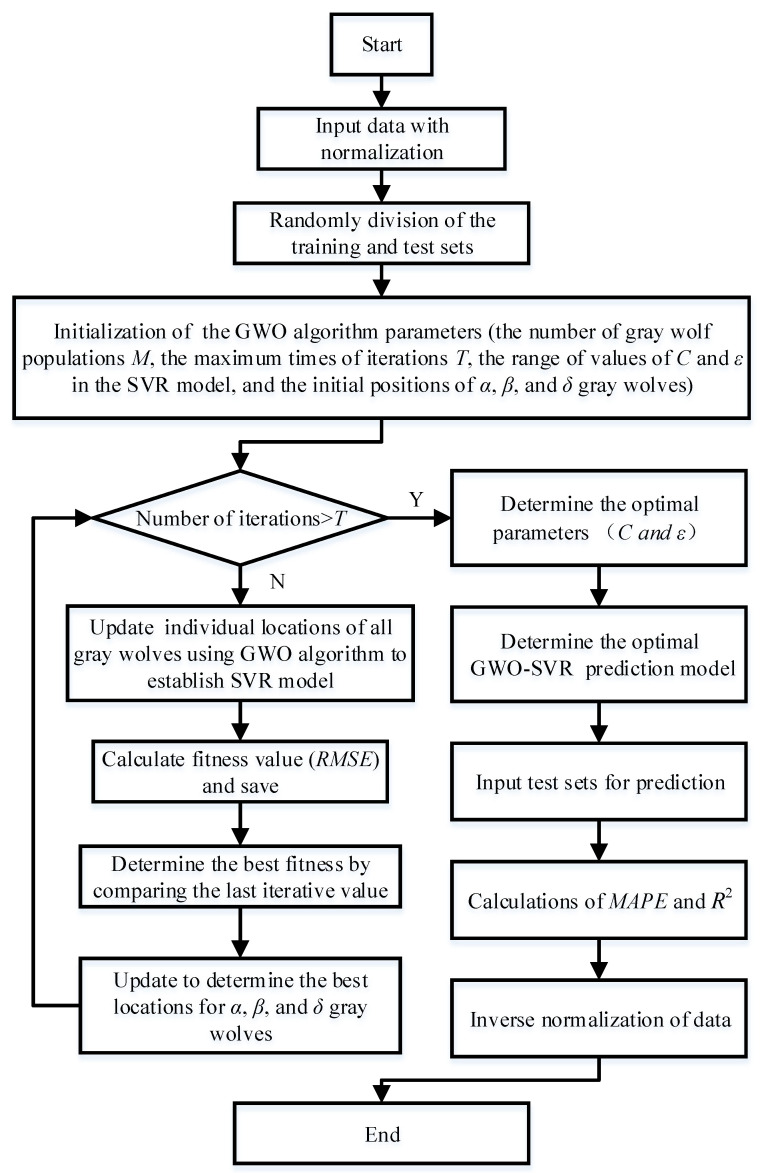
Flow chart of the GWO-SVM surface roughness prediction model.

In this paper, the experimental results in Table 9 are used as the data source to construct a GWO-SVR prediction model for the surface roughness of LAM SiC ceramics using the MATLAB software in two processes. Firstly, the SVR parameters *C* and *ε* are optimized using the GWO algorithm, and then the optimized SVR is used for roughness prediction. Figure 7 shows the flow chart of the GWO-SVM surface roughness prediction model, with the key steps of the optimization as follows:

(1) The dimension of laser power is different from that of other process parameters. The process parameters with larger dimension will dominate, and the prediction results of surface roughness are more sensitive to the changes of process parameters with larger values. In order to improve the accuracy of the prediction model, the interval scaling method is used to normalize the original input sample space, and the process parameters of different dimensions are converted to [0, 1] using Equation (17):
  (17) $$y=2\cdot \frac{x-x_{\min}}{x_{\max}-x_{\min}}-1$$
  where *y* is the normalized value of the output; *x* is the input variable value; $x_{\max}$ and $x_{\min}$ are the maximum and minimum values of input variables, respectively.
(2) The 25 sets of test data are numbered, 20 of the sets are randomly selected as training samples, and the remaining 5 sets of data are used as test samples.
(3) In this paper, the number of initialized wolf groups M is 20, the number of iterations T is 200, and the parameters *C* and *ε* to be optimized take values in the range of 0.001~100.
(4) GWO takes the root mean square error (*RMSE*) as the fitness function (Equation (18)) to evaluate the optimal parameters of the SVR. The optimal internal parameters *C* and *ε* of the prediction model can be obtained by optimizing, and therefore, the value of the root mean square error (*RMSE*) of the training samples of the SVR algorithm is minimized:
  (18) $$RMSE=\frac{\sqrt{\sum_{i=1}^{20}{\left(y_{i}-y_{Ra}\right)}^{2}}}{20}$$
(5) The constructed SVR prediction model is used to establish the relationship between each process parameter and the surface roughness (*Ra*) value through the data in the training sample, and the constructed regression model is evaluated based on the coefficient of determination (*R^2^*) and the mean absolute percentage error (MAPE) of the test sample, as shown in Equations (19) and (20). The *R^2^* is used to evaluate the degree of fitting of the regression model to the sample space; the *MAPE* is used to evaluate the volatility of the predicted data. The closer the value of *R^2^* is to one and the closer the value of *MAPE* is to zero, the better the fitting of the predicted data to the experimental data, the better the reliability, and the more accurate the model prediction:
  (19) $$R^{2}=1-\frac{\sum_{i=1}^{5}{\left(y_{Ra}-y_{i}\right)}^{2}}{\sum_{i=1}^{5}{\left(y_{i}-\bar{y}\right)}^{2}}$$
  (20) $$MAPE=\frac{\sum_{i=1}^{5}\left|\frac{y_{i}-y_{Ra}}{y_{i}}\right|}{5}\times 100\%$$

The value of *R*^2^ is used to evaluate the degree of fitting of the regression model to the sample space; the value of *MAPE* is used to evaluate the volatility of the predicted data. The closer the value of *R*^2^ is to one and the closer the value of *MAPE* is to zero, the better the fitting of the predicted data to the experimental data, the better the reliability, and the more accurate the model prediction.

## 4. Results and Discussion

### 4.1. Analysis of Experimental and Predicted Results

**Figure 8:**
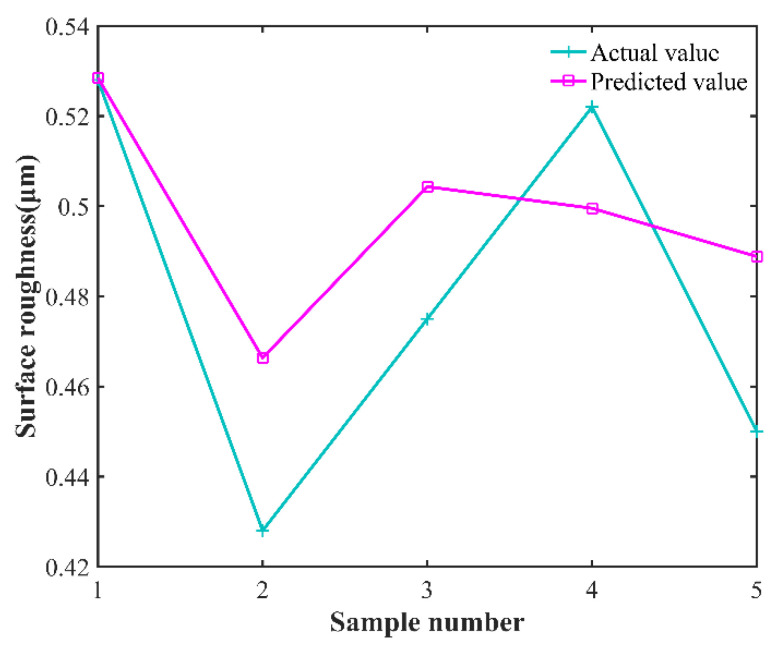
SVR model predicted curve versus actual curve.

**Figure 9:**
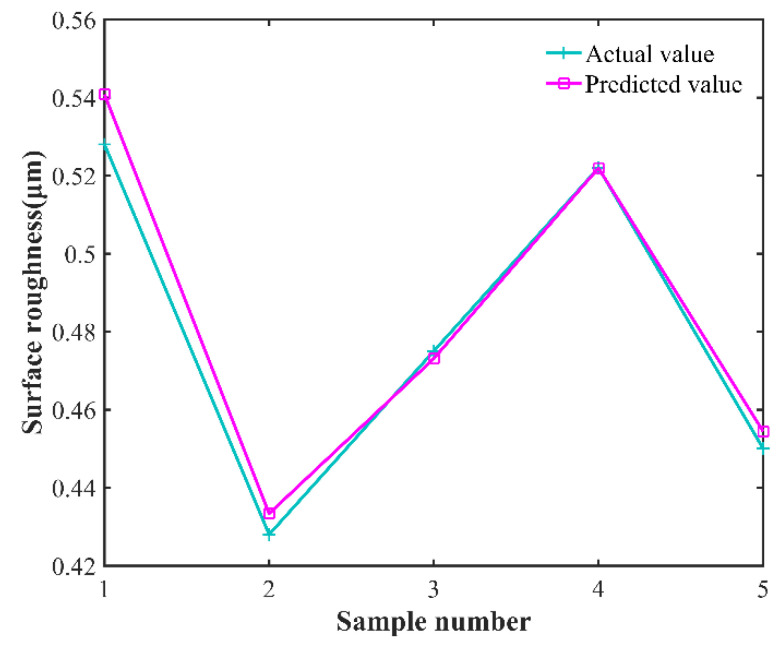
GWO-SVR model predicted curve versus actual curve.

**Figure 10:**
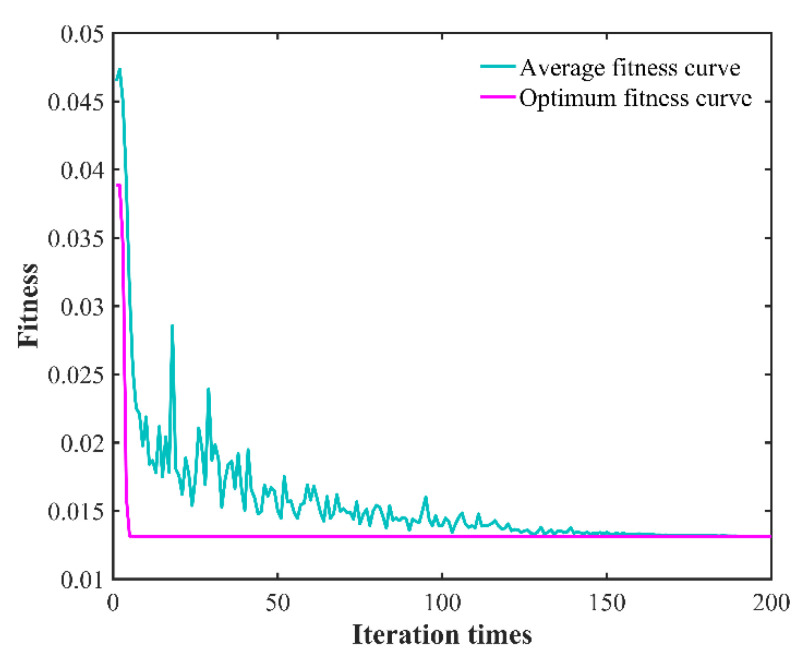
*RMSE* fitness curve of variation.

After the GWO-SVR prediction model is constructed, the predicted and experimental results of the test samples before and after model optimization are analyzed. In order to show the comparative differences more clearly and to reflect the optimization effect, the line plot of the predicted results and experimental results before and after the model optimization as well as the change in fitness curve are plotted, as shown in Figure 8, Figure 9 and Figure 10, respectively. The predicted and experimental results for the test samples are compared, and are shown in Table 10.

Table 10 shows that the average relative error of the optimized model decreased from 5.325% to 2.624; the coefficient of determination increased from 0.738564 to 0.98676; the fitness value decreased from 0.0295 to 0.0065; and the average absolute error decreased from 5.6289% to 2.6639%. The GWO-SVR prediction model has better prediction accuracy and reliability, and can achieve accurate prediction of surface roughness under different process parameters, providing a new method for rapid assessment of surface roughness of laser-assisted turning of SiC ceramics.

### 4.2. Optimization of Process Parameters and Validation

In order to obtain better surface roughness and to improve the surface finish of the workpiece, it is necessary to determine the optimal combination of process parameters. Therefore, the GWO-SVR prediction model is used as a fitness function to invoke the GWO algorithm for process parameter optimization. The optimization ranges of the process parameters are shown in Table 11.

**Figure 11:**
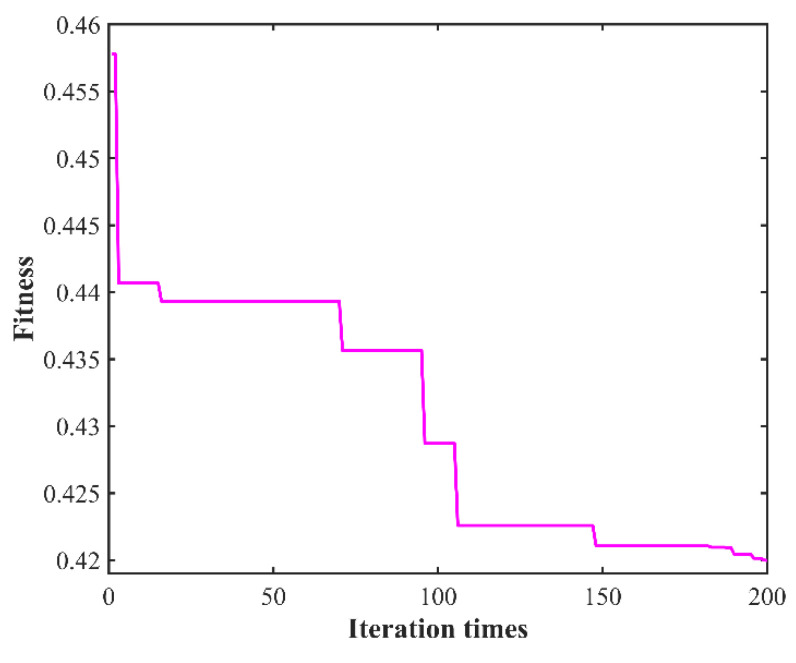
Curve of fitness variation.

After 200 iterations, the optimum surface roughness *Ra* value of 0.41997 μm is obtained, which corresponds to the following combination of process parameters: laser power of 210.3025 W, rotational speed of 1639.4165 r/min, feed rate of 2.5808 mm/min, and cutting depth of 0.1421 mm. The fitness curve is shown in Figure 11.

**Figure 12:**
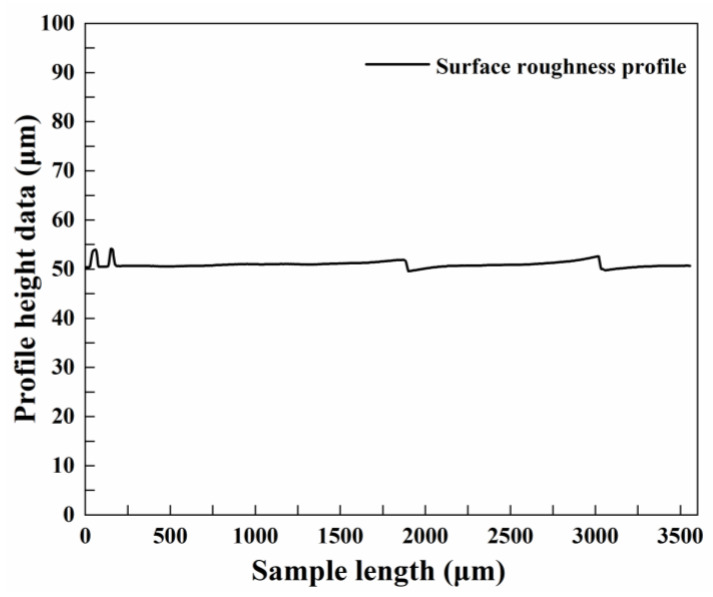
Curve of surface roughness profile.

Due to the limitations of the process parameter adjustment of the experimental equipment, the processing cannot be carried out exactly according to the theoretically optimized process parameters, which needs to be adjusted. The adjusted process parameter combination is as follows: laser power of 210 W, rotational speed of 1639 r/min, feed rate of 2.58 mm/min, and cutting depth of 0.142 mm. In order to ensure the accuracy of the experimental results, the experiments are conducted three times under the combined conditions of the adjusted process parameters, and the results are shown in Table 12. Figure 12 shows the surface roughness profile curve of the workpiece for Experiment No. 1 in Table 12.

**Figure 13:**
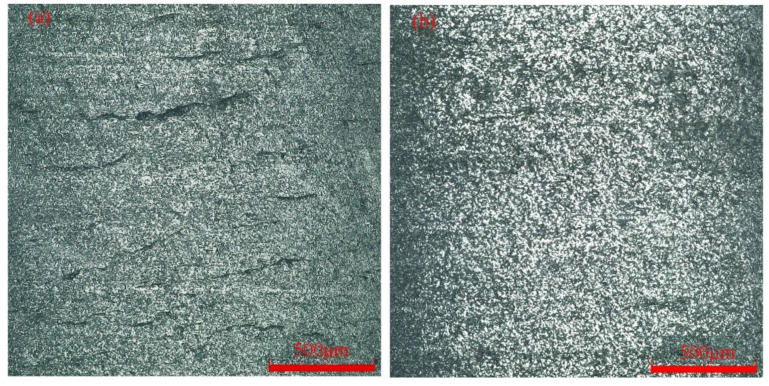
Surface morphology of different specimens: (**a**) No heating of laser; (**b**) Optimized specimen.

The maximum surface roughness of Specimen 18 in Table 9 was 0.538 μm, and this was compared with the surface morphology of the workpiece after the global optimization experiment. Figure 13 shows the surface morphology of different specimens. Figure 13a shows the surface morphology of a specimen in the conventional turning method without laser heating. There are a lot of microcracks on the surface of the workpiece, which affect the fatigue strength and service life of the workpiece, and therefore, the workpiece cannot be applied to engineering. Figure 13b shows the surface morphology of the optimized specimen. The surface texture is more uniform and there are no cracks or thermal damages on the surface of the workpiece, which significantly improves the surface quality and makes it suitable for engineering applications.

Figure 13 shows that after the optimization of the process parameters, the surface texture of the machined surface is more regular and uniform, with almost no cracks, pits, or thermal damage, and the surface quality has been significantly improved. The GWO algorithm was invoked to perform a global search for optimization of the GWO-SVR prediction model to quickly determine the optimal combination of process parameters for LAM of SiC ceramics, providing an efficient method for future targeted searches for optimal surface roughness.

## 5. Conclusions

In this paper, we describe the processing principle and material removal mechanism for LAM of SiC ceramics. The GWO-SVR machine learning algorithm model is used to predict the surface roughness and to optimize the process parameters, and the minimum value of surface roughness within the process parameters is determined using the GWO algorithm. The micromorphology and the surface quality of SiC ceramic workpieces are significantly improved. The details are summarized as follows.

1. Based on the processing principle of laser-assisted machining of SiC ceramics and the material removal mechanism, the effects of laser power, rotational speed, feed rate, and cutting depth on surface roughness are investigated by performing single-factor experiments. The surface roughness *Ra* values show a trend of decreasing, and then increasing with an increase in the level of each factor in the study range. The values of surface roughness are minimums of 0.45 μm, 0.461 μm, 0.428 μm, and 0.461μm, when the single-factor levels are 215 W, 1620 r/min, 2.5 mm/min, and 0.15 mm, respectively.
2. The SVR prediction model with laser power, rotational speed, feed rate, and cutting depth as input values and surface roughness as output value is constructed through single-factor as well as orthogonal experiments, and the GWO algorithm is used to optimize the SVR prediction model. The average relative error of the GWO-SVR surface roughness prediction model decreases to 2.624%, the coefficient of determination *R*^2^ increases to 0.98676, the best RMSE of fitness decreases to 0.0065, and the mean absolute percentage error (MAPE) decreases to 2.6639%. The prediction accuracy and reliability are improved, and the accurate prediction of surface roughness of laser-assisted machining SiC ceramics is achieved.
3. Global optimization is carried out by using the constructed GWO-SVR prediction model based on the GWO algorithm. When the laser power is 210.3025 W, the rotational speed is 1639.4165 r/min, the feed rate is 2.5808 mm/min, and the cutting depth is 0.1421 mm, the minimum surface roughness *Ra* value is 0.41997 μm. The surface roughness error between the actual and predicted value is less than 1.91%, the surface texture of the workpiece is regular and more uniform, and the surface quality and micromorphology are significantly improved.

## Figures and Tables

**Table 1:** Physical and mechanical property parameters.

Items	SiC
Elastic modulus (GPa)	290
Vickers hardness (kgf·mm^−2^)	2100
Compressive strength (MPa)	3000
Fracture toughness (MPa·m^1/2^)	4
Thermal expansion coefficient × 10^−6^/℃	4.5
Thermal conductivity (W/mK)	80
Melting point (K)	3100
Specific heat capacity (J/kgK)	1100
Density (g/cm^3^)	3.15

Note: the parameters listed in Table 1 are supplied by manufacturer.

**Table 2:** Experimental parameters.

No.	Process Parameter	Value Range
1	Laser power (W)	185~225
2	Rotational speed (r/min)	1440~1800
3	Feed speed (mm/min)	2~4
4	Cutting depth(mm)	0.1~0.2

**Table 3:** Experimental results at different laser powers.

No.	Laser Power (W)	Rotational Speed (r/min)	Feed Speed (mm/min)	Cutting Depth (mm)	Surface Roughness (μm)
1	185	1620	3	0.15	0.51
2	195	1620	3	0.15	0.492
3	205	1620	3	0.15	0.461
4	215	1620	3	0.15	0.45
5	225	1620	3	0.15	0.475

**Table 4:** Experimental results at different rotational speeds.

No.	Laser Power (w)	Rotational Speed (r/min)	Feed Speed (mm/min)	Cutting Depth (mm)	Surface Roughness (μm)
1	205	1440	3	0.15	0.527
2	205	1530	3	0.15	0.496
3	205	1620	3	0.15	0.461
4	205	1710	3	0.15	0.48
5	205	1800	3	0.15	0.522

**Table 5:** Experimental results at different feed speeds.

No.	Laser Power (W)	Rotational Speed (r/min)	Feed Speed (mm/min)	Cutting Depth (mm)	Surface Roughness (μm)
1	205	1620	2	0.15	0.47
2	205	1620	2.5	0.15	0.428
3	205	1620	3	0.15	0.461
4	205	1620	3.5	0.15	0.521
5	205	1620	4	0.15	0.524

**Table 6:** Experimental results at different cutting depths.

No.	Laser Power (W)	Rotational Speed (r/min)	Feed Speed (mm/min)	Cutting Depth (mm)	Surface Roughness (μm)
1	205	1620	3	0.1	0.505
2	205	1620	3	0.125	0.47
3	205	1620	3	0.15	0.461
4	205	1620	3	0.175	0.498
5	205	1620	3	0.2	0.531

**Table 7:** Factor and level table.

No.	Laser Power (W)	Rotational Speed (r/min)	Feed Speed (mm/min)	Cutting Depth (mm)
1	185	1440	2	0.1
2	205	1620	3	0.15
3	225	1800	4	0.2

**Table 8:** Results of the orthogonal experiment.

No.	Laser Power (W)	Rotational Speed (r/min)	Feed Speed (mm/min)	Cutting Depth (mm)	Surface Roughness (μm)
1	185	1440	2	0.1	0.538
2	185	1620	3	0.15	0.51
3	185	1800	4	0.2	0.53
4	205	1440	3	0.2	0.528
5	205	1620	4	0.1	0.536
6	205	1800	2	0.15	0.496
7	225	1440	4	0.15	0.528
8	225	1620	2	0.2	0.525
9	225	1800	3	0.1	0.495

**Table 9:** Experimental results at different process parameters.

No.	Laser Power (W)	Rotational Speed (r/min)	Feed Speed (mm/min)	Cutting Depth (mm)	Surface Roughness (μm)
1	185	1620	3	0.15	0.51
2	195	1620	3	0.15	0.492
3	205	1620	3	0.15	0.461
4	215	1620	3	0.15	0.45
5	225	1620	3	0.15	0.475
6	205	1440	3	0.15	0.527
7	205	1530	3	0.15	0.496
8	205	1710	3	0.15	0.48
9	205	1800	3	0.15	0.522
10	205	1620	2	0.15	0.47
11	205	1620	2.5	0.15	0.428
12	205	1620	3.5	0.15	0.521
13	205	1620	4	0.15	0.524
14	205	1620	3	0.1	0.505
15	205	1620	3	0.125	0.47
16	205	1620	3	0.175	0.498
17	205	1620	3	0.2	0.531
18	185	1440	2	0.1	0.538
19	185	1800	4	0.2	0.53
20	205	1440	3	0.2	0.528
21	205	1620	4	0.1	0.536
22	205	1800	2	0.15	0.496
23	225	1440	4	0.15	0.528
24	225	1620	2	0.2	0.525
25	225	1800	3	0.1	0.495

**Table 10:** Comparisons of predicted and experimental results of test samples.

No.	Laser Power (W)	Rotationa Speed (r/min)	Feed Speed (mm/min)	Cuttin Depth (mm)	Actua Value (μm)	SVR		GWO-SVR	
						Predicte Value (μm)	Relativ Error (%)	Predicte Value (μm)	Relativ Error (%)
1	205	1440	3	0.2	0.528	0.5285	0.095	0.5436	2.87
2	205	1620	2.5	0.15	0.428	0.4663	8.28	0.4374	2.15
3	225	1620	3	0.15	0.475	0.5043	5.81	0.4850	2.06
4	205	1800	3	0.15	0.522	0.4995	4.50	0.5074	2.88
5	215	1620	3	0.15	0.45	0.4888	7.94	0.4647	3.16
*R* ^2^						0.738564		0.98676	
*RMSE*						0.0295		0.0065	
*MAPE*/%						5.6289		2.6639	

**Table 11:** Process parameter ranges.

Process Parameter	Range
Laser power (W)	[185, 225]
Rotational speed (r/min)	[1440, 1800]
Feed speed (mm/min)	[2, 4]
Cutting depth (mm)	[0.1, 0.2]

**Table 12:** Optimized experimental results.

No.	Laser Power (W)	Rotational Speed (r/min)	Feed Speed (mm/min)	Cutting Depth (mm)	Actual Value (μm)	Predicted Value (μm)	Relative Error (%)
1	210	1639	2.58	0.142	0.418	0.41997	0.47
2	210	1639	2.58	0.142	0.427	0.41997	1.67
3	210	1639	2.58	0.142	0.428	0.41997	1.91

## Data Availability

Not Applicable.
